# Supplementary material for: A Phylogenomic Assessment of Processes Underpinning Convergent Evolution in Open-Habitat Chats
Source: Mol Biol Evol. 2022 Dec 29;40(1):msac278. doi: 10.1093/molbev/msac278 (PMC10161543; doi:10.1093/molbev/msac278)
Supplement: msac278_Supplementary_Data [file msac278_supplementary_data.pdf]

Supplementary data

Table S1 | Sampling and sequence data information.

Taxon	Collection, no.[a]	Tissue	Library Type	Mapping %	Coverage
Campicoloides bifasciatus	MVZ, RSA073,	Muscle	Illumina DNA Prep Kit	99.1	8.5
Emarginata schlegelii	FMNH, 453197,	Muscle	Illumina DNA Prep Kit	99.1	9.5
Emarginata sinuata	UWMB, 95470	Muscle	ThruPLEX DNA-Seq Kit	96.7	9.7
Myrmecocichla aethiops	MVZ, 129113	Toepad	ACCEL-NGS 1S DNA Library Prep Kit	93.3	6.8
Myrmecocichla arnotti	FMNH, 468111	Toepad	Illumina DNA Prep Kit	99.3	12.8
Myrmecocichla formicivora	MVZ, RSA205	Muscle	Illumina DNA Prep Kit	98.6	8.3
Myrmecocichla melaena	A1153	Blood	ThruPLEX DNA-Seq Kit	98.8	13.3
Myrmecocichla monticola	NMBE, 1043860	Toepad	ACCEL-NGS 1S DNA Library Prep Kit	94.3	7.6
Myrmecocichla nigra	NRM, 570041	Toepad	ThruPLEX DNA-Seq Kit	94.5	7.0
Myrmecocichla tholloni	YPM, ORN95640	Toepad	ThruPLEX DNA-Seq Kit	92.1	7.3
Oenanthe (C.) dubia	FMNH, 83201	Toepad	ACCEL-NGS 1S DNA Library Prep Kit	93.0	6.8
Oenanthe (C.) familiaris	MVZ, GO866	Blood	Illumina DNA Prep Kit	99.4	13.3
Oenanthe (C.) fusca	YPM, ORN011707	Toepad	ThruPLEX DNA-Seq Kit	86.4	4.7
Oenanthe (C.) melanura	A1203	Blood	ThruPLEX DNA-Seq Kit	98.8	15.4
Oenanthe (C.) scotocerca	LACM, 61131	Toepad	ACCEL-NGS 1S DNA Library Prep Kit	96.6	8.3
Oenanthe (M.) albifrons clericalis	NRM, 558941	Toepad	ThruPLEX DNA-Seq Kit	95.4	6.3
Oenanthe (M.) albifrons frontalis	KU, 115365	Muscle	ThruPLEX DNA-Seq Kit	98.5	12.7
Oenanthe albonigra	IR-KIL-010	Blood	ThruPLEX DNA-Seq Kit	99.2	12.9

Taxon	Collection, no.[a]	Tissue	Library Type	Mapping %	Coverage
Oenanthe bottae frenata	NRM, 558917	Toepad	ThruPLEX DNA-Seq Kit	97.0	6.0
Oenanthe chrysopygia	IR-FIR-002	Blood	ThruPLEX DNA-Seq Kit	98.9	11.5
Oenanthe cypriaca	19e	Blood	Chromium Genome Library kit	99.8	40.6
Oenanthe deserti	MO-BOULMANE-2013	Blood	ThruPLEX DNA-Seq Kit	99.3	12.6
Oenanthe finschii	IR-ESF-004	Blood	ThruPLEX DNA-Seq Kit	98.9	8.7
Oenanthe halophila	3Y42902	Blood	Illumina DNA PCR-free	99.6	17.4
Oenanthe heuglinii	ZFMK, H.II.16p2.α	Dry skin	ACCEL-NGS 1S DNA Library Prep Kit	93.6	5.7
Oenanthe hispanica	E-GUI-013	Blood	Chromium Genome Library kit	99.8	15.5
Oenanthe isabellina	GR-LES-001	Blood	ThruPLEX DNA-Seq Kit	99.2	9.9
Oenanthe leucopyga leucopyga	A1137	Blood	ThruPLEX DNA-Seq Kit	98.8	10.7
Oenanthe leucura leucura	E-MAT-2012	Blood	ThruPLEX DNA-Seq Kit	99.0	9.5
Oenanthe lugens lugens	9b	Blood	ThruPLEX DNA-Seq Kit	98.7	22.0
Oenanthe lugens persica	ZMUC, 137759	Muscle	ThruPLEX DNA-Seq Kit	98.6	10.4
Oenanthe lugentoides lugentoides	NHMUK, 1965.M.12140	Toepad	ThruPLEX DNA-Seq Kit	96.9	4.6
Oenanthe lugentoides boscaweni	NHMUK, 1977.M.21.36	Toepad	ThruPLEX DNA-Seq Kit	97.2	6.8
Oenanthe lugubris lugubris	A1129	Blood	ThruPLEX DNA-Seq Kit	99.0	12.5
Oenanthe lugubris vauriei	AMNH, 461151	Toepad	ThruPLEX DNA-Seq Kit	95.9	5.3
Oenanthe melanoleuca	IT-GRA-006	Blood	Chromium Genome Library kit	99.7	12.6
Oenanthe moesta	A1109	Blood	ThruPLEX DNA-Seq Kit	98.8	12.0
Oenanthe monacha	A1174	Blood	ThruPLEX DNA-Seq Kit	99.3	17.2

Taxon	Collection, no.[a]	Tissue	Library Type	Mapping %	Coverage
Oenanthe oenanthe	GEO-VAR-011	Blood	ThruPLEX DNA-Seq Kit	99.1	12.2
Oenanthe phillipsi	YPM, ORN035210	Toepad	ThruPLEX DNA-Seq Kit	95.6	7.9
Oenanthe picata capistrata	ZMUC, 29495	Toepad	ThruPLEX DNA-Seq Kit	96.7	5.3
Oenanthe picata opistholeuca	ZMUC, 29578	Toepad	ThruPLEX DNA-Seq Kit	97.1	5.2
Oenanthe picata picata	IR-TAN-005	Blood	ThruPLEX DNA-Seq Kit	98.8	14.2
Oenanthe pileata	TCWC, 15606	Muscle	Illumina DNA Prep Kit	98.9	13.4
Oenanthe pleschanka	CN-XS-006	Blood	Chromium Genome Library kit	99.8	15.1
Oenanthe seebohmi	KA69373	Blood	ThruPLEX DNA-Seq Kit	99.2	11.2
Oenanthe warriae	12c	Blood	ThruPLEX DNA-Seq Kit	98.7	13.4
Oenanthe xanthoprymna	NHMO, 15188	Blood	ThruPLEX DNA-Seq Kit	99.0	12.9
Pinarochroa sordida	YPM, ORN80066	Toepad	ThruPLEX DNA-Seq Kit	95.4	6.5
Thamnolaea cinnamomeiventris	NRM, 20086147	Muscle	ThruPLEX DNA-Seq Kit	98.6	15.9

[a] AMNH; American Museum of Natural History; NHMUK: Natural History Museum, Tring; FMNH: Field Museum of Natural History; LACM: Natural History Museum of Los Angeles County; MVZ: Museum of Vertebrate Zoology, UC Berkeley; NHMO: Natural History Museum, University of Oslo; NRM: Naturhistoriska riksmuseet, Stockholm; TCWC: Texas A&M University Biodiversity Research and Teaching Collections; UWBM: University of Washington Burke Museum; YPM: Yale Peabody Museum; ZMUC: Zoological Museum, Natural History Museum of Denmark; ZFMK: Zoologisches Forschungsmuseum König. Samples for which no institution is indicated are part of the research group’s collection.

Table S2 | Number of loci across the genome using different filtering settings for 10 kb non-overlapping windows: minimum read depth (DP), minimum percentage of the window covered by data (PW), and missing data per site (MD).

Filtering setting	Number of total windows	Number of no intra-locus recombination windows	Number of no intra-locus and free inter-locus recombination windows
DP=1, PW=50%, MD=15%	29,730	10,051	6,791
DP=5, PW=50%, MD=15%	19,062	6,701	5,788
DP=1, PW=50%, MD=5%	26,030	9,600	6,476
DP=1, PW=80%, MD=10%	22,652	6,970	5,267

Table S3 | Selection of the best models among the maximum pseudo-likelihood networks in phyloNet 3.6.9.

**Complex**	**Gene trees**	**Inferred admixtures**	**Likelihood**	**BIC**
O. picata	7,310	0	-33,633.58	67,278.88
		**1**	**-33,559.56**	**67,131.21**
		2	-33,569.10	67,150.56
		3	-33,617.49	67,247.56
		4	-33,979.37	67,971.48
		5	-33,982.37	67,977.62
O. hispanica	7,335	0	-33,326.92	66,665.57
		1	-33,180.89	66,373.86
		**2**	**-33,153.96**	**66,320.28**
		3	-33,265.29	66,543.14
		4	-33,276.89	66,566.51
		5	-33,637.19	67,287.26
O. lugens	,7323	0	-86,457.1	17,2926.3
		1	-85,643.3	17,1299.0
		**2**	**-85,014.9**	**17,0042.3**
		3	-86,498.8	17,3010.4
		4	-86,574.7	17,3162.3
		5	-86,095.2	17,2203.3

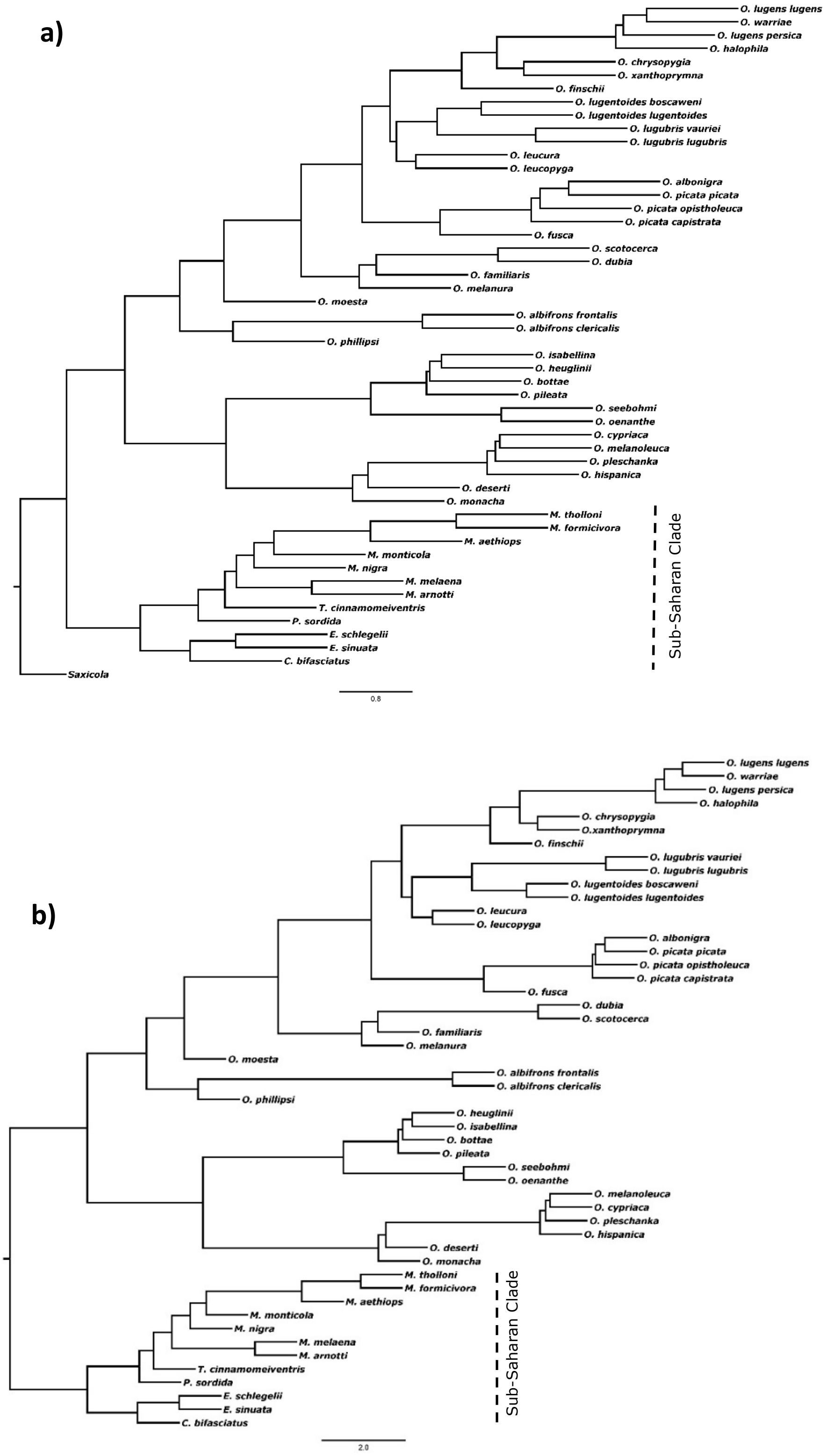

Figure S1 | Genome-wide multispecies coalescent species tree of open-habitat chats estimated in ASTRAL-III. All nodes have 1.0 local posterior probabilities. a) Species tree based on 2,091 BUSCOs, using *Saxicola* as an outgroup. b) Species tree based on 5,788 loci (non-overlapping 10 kb windows across the genome) using the sub-Saharan clade as an outgroup.

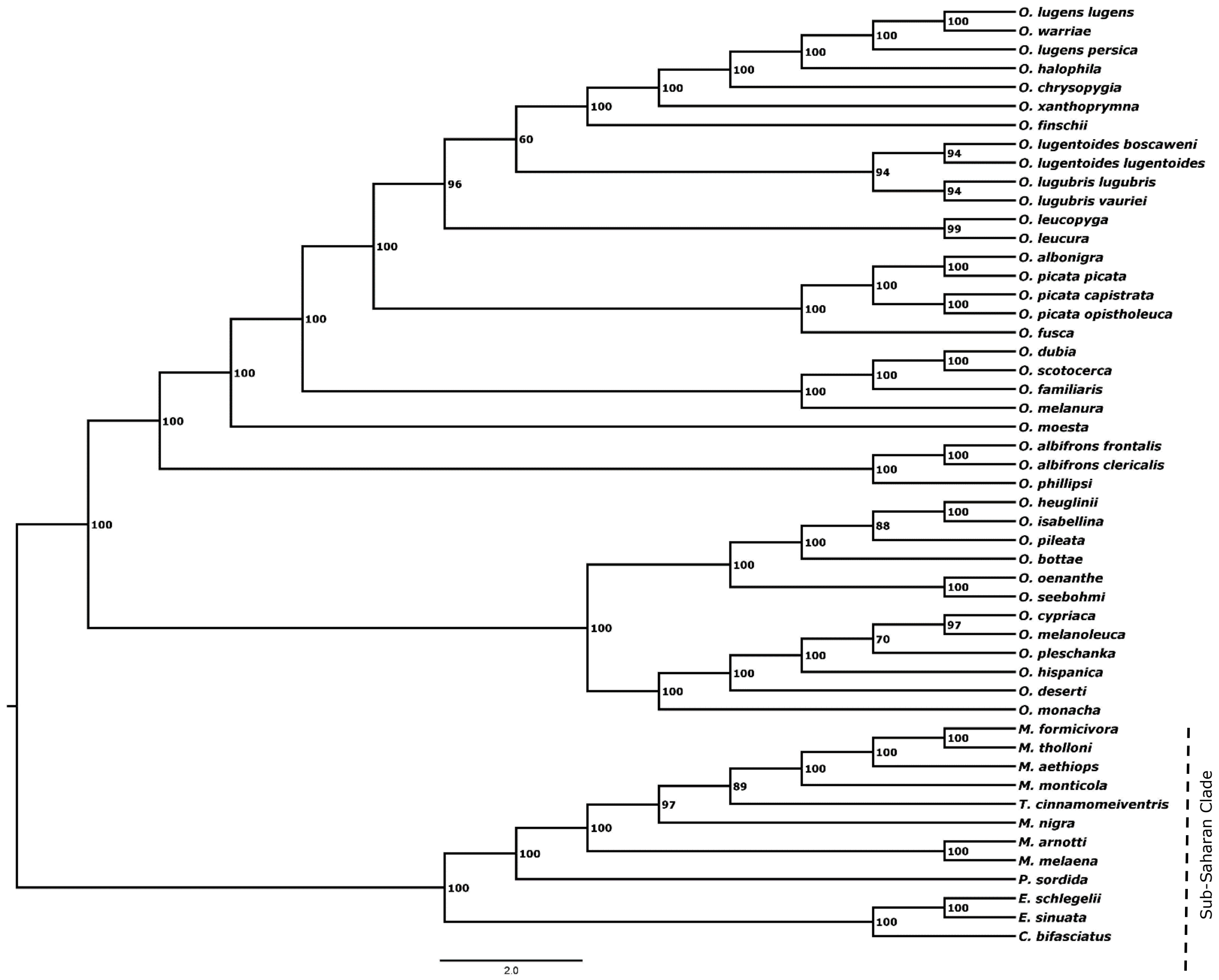

Figure S2 | SNP-based multispecies coalescent species tree of open-habitat chats inferred with SVDquartets in PAUP* 4. Maximum likelihood bootstrap support values are shown above branches.

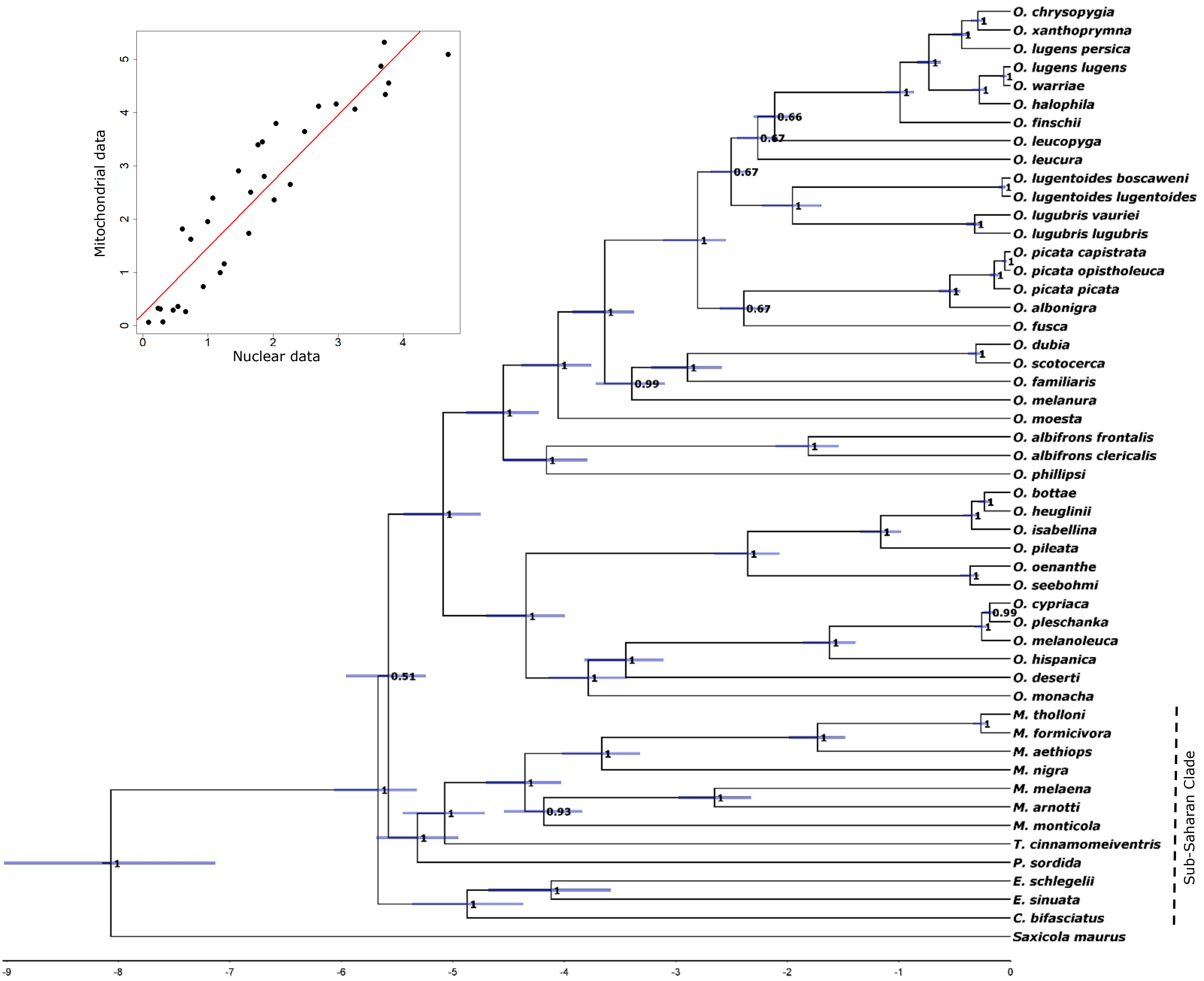

Figure S3 | Time-calibrated maximum clade credibility tree of the BEAST 2.6.6 analyses based on 13 protein-coding mitochondrial genes of open-habitat chats. Blue bars represent the 95% highest posterior density (HPD) distributions for the estimated divergence times. Posterior probabilities are indicated at nodes. The correlation between the dating based on the nuclear (BUSCOs) and mitochondrial data is shown at the top left (Pearson's r=0.93, p<0.001).

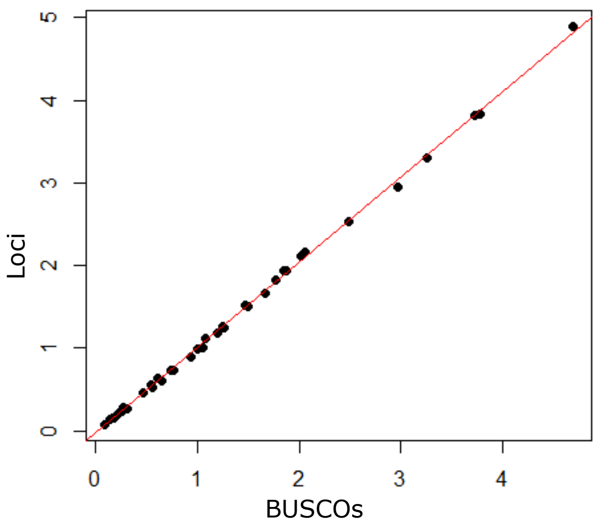

Figure S4 | Correlation between the time calibration based on 1.8 Mb high confidence BUSCOs and 3.8 Mb high confidence loci (10 kb non-overlapping windows across the genome) using RelTime-ML implemented in MEGA 11 (Pearson's r=0.99, p<2.2e-16).

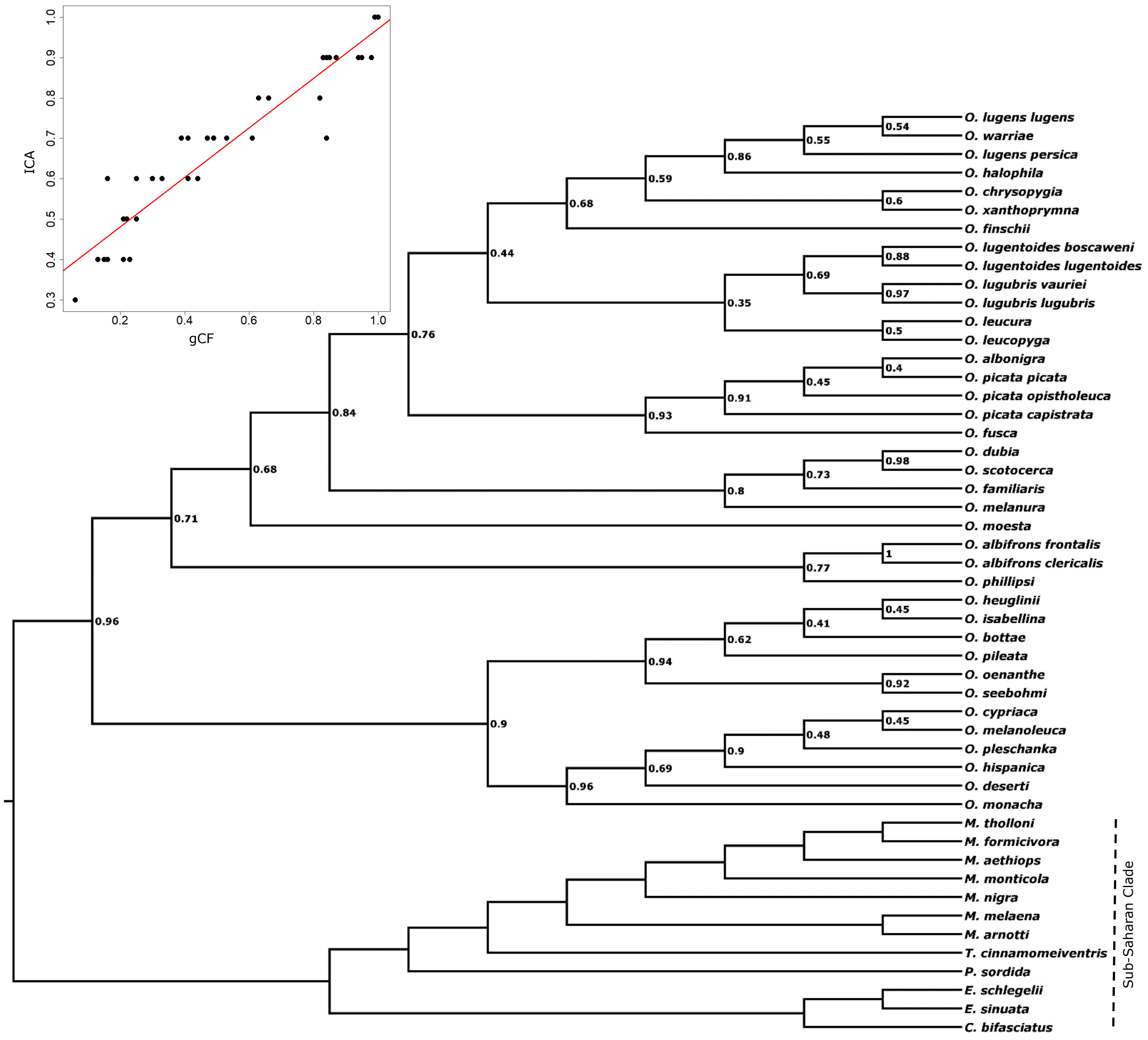

Figure S5 | Genome-wide multispecies coalescent species tree of open-habitat chats estimated in ASTRAL-III. ICA values for 29,730 maximum-likelihood gene trees are shown for each node. The high correlation between the gCF values calculated in IQ-TREE 2.1.2 and ICA values calculated in PhyParts 0.0.1 is shown at the top left (Pearson's r=0.94, p<0.001).
